# Effects of the PARP Inhibitor Olaparib on the Response of Human Peripheral Blood Leukocytes to Bacterial Challenge or Oxidative Stress

**DOI:** 10.3390/biom12060788

**Published:** 2022-06-04

**Authors:** Sidneia Sousa Santos, Milena Karina Coló Brunialti, Larissa de Oliveira Cavalcanti Peres Rodrigues, Ana Maria Alvim Liberatore, Ivan Hong Jun Koh, Vanessa Martins, Francisco Garcia Soriano, Csaba Szabo, Reinaldo Salomão

**Affiliations:** 1 Department of Medicine, Division of Infectious Diseases, Escola Paulista de Medicina, Federal University of São Paulo (EPM/UNIFESP), São Paulo 04023, Brazil; sidneia.sousa@unifesp.br (S.S.S.); milena.brunialti@unifesp.br (M.K.C.B.); larissa.cavalcanti@unifesp.br (L.d.O.C.P.R.); 2 Discipline of Operative Technique and Experimental Surgery, Department of Surgery, Federal University of São Paulo (EPM/UNIFESP), São Paulo 04023, Brazil; analiber@terra.com.br (A.M.A.L.); ivankoh@terra.com.br (I.H.J.K.); 3 Chair of Pharmacology, Faculty of Science and Medicine, University of Fribourg, 1700 Fribourg, Switzerland; vanessa.martins@unifr.ch; 4 Laboratory of Medical Research, Faculty of Medicine, University of São Paulo, São Paulo 05403, Brazil; gsoriano@usp.br

**Keywords:** PARP1, DNA damage, olaparib, inflammation, NAD^+^, ATP, sepsis, repurposing

## Abstract

Prior studies demonstrate the activation of poly-(ADP-ribose) polymerase 1 (PARP1) in various pathophysiological conditions, including sepsis. We have assessed the effect of olaparib, a clinically used PARP1 inhibitor, on the responses of human peripheral blood leukocytes (PBMCs) obtained from healthy volunteers in response to challenging with live bacteria, bacterial lipopolysaccharide (LPS), or oxidative stress (hydrogen peroxide, H_2_O_2_). The viability of PBMCs exposed to olaparib or to the earlier generation PARP inhibitor PJ-34 (0.1–1000 µM) was monitored using Annexin V and 7-aminoactinomycin D. To evaluate the effects of olaparib on the expression of PARP1 and its effects on protein PARylation, PBMCs were stimulated with *Staphylococcus aureus* with or without olaparib (1–10 μM). Changes in cellular levels of nicotinamide adenine dinucleotide (NAD^+^) and adenosine triphosphate (ATP), as well as changes in mitochondrial membrane potential (MMP), were measured in PBMCs exposed to H_2_O_2_. Bacterial killing was evaluated in PBMCs and polymorphonuclear leukocytes (PMNs) incubated with *S. aureus*. Cytokine production was measured in supernatants using a cytometric bead array. Reactive oxygen species (ROS), nitric oxide (NO) production, and phagocytic activity of monocytes and neutrophils were measured in whole blood. For ROS and NO production, samples were incubated with heat-killed *S. aureus*; phagocytic activity was assessed using killed *Escherichia coli* conjugated to FITC. Olaparib (0.1–100 µM) did not adversely affect lymphocyte viability. Olaparib also did not interfere with PARP1 expression but inhibits *S. aureus*-induced protein PARylation. In cells challenged with H_2_O_2_, olaparib prevented NAD^+^ and ATP depletion and attenuated mitochondrial membrane depolarization. LPS-induced production of TNF-α, MIP-1α, and IL-10 by PBMCs was also reduced by olaparib. Monocytes and neutrophils displayed significant increases in the production of ROS and NO after stimulation with *S. aureus* and phagocytic (*E. coli*) and microbicidal activity, and these responses were not suppressed by olaparib. We conclude that, at clinically relevant concentrations, olaparib exerts cytoprotective effects and modulates inflammatory cytokine production without exerting adverse effects on the cells’ ability to phagocytose or eradicate pathogens. The current data support the concept of repurposing olaparib as a potential experimental therapy for septic shock.

## 1. Introduction

Poly-(ADP-ribose) polymerase 1 (PARP1) is a constitutive enzyme that, in response to genotoxic agent-induced single-stranded DNA damage, catalyzes a reaction in which the ADP-ribose portion of nicotinamide adenine dinucleotide (NAD^+^) is transferred to an amino acid receptor to produce poly (ADP-ribose) polymers through the process of PARylation [1]. PARylation can be removed through the action of PAR glycohydrolase (PARG) or PAR hydrolase (ARH3), which break down PAR into free ADP-ribose monomeric or oligomeric units [1,2]. PARP1 overactivation consumes large amounts of NAD^+^, an essential molecule that acts as a substrate for various enzymes with crucial roles in metabolism, aging, cell survival, and ATP production [3]. The PAR polymers produced during PARP1 activation can affect several different cellular processes in various cellular compartments. PAR serves as a key molecule that triggers a cell death mechanism known as parthanatos, which is distinct from apoptosis and necrosis and does not depend on caspases. PAR polymers can also induce the mitochondrial release of apoptosis-inducing factor (AIF). AIF translocates to the nucleus, where it causes chromatin condensation and DNA fragmentation [1,2,3,4]. In the extracellular space, PAR may act as a pro-inflammatory signal to macrophages, inducing phagocytosis and stimulating cytokine secretion by binding to Toll-like receptors (TLR) 2 and 4 [5]. PARylation can also enhance the activation of a pro-inflammatory response in macrophages; some of these responses have been attributed to the interaction of PARP1 and nuclear factor-kappa B (NF-κB), which culminates in the transcription of various genes including those related to the production of cytokines [1,6]. PARP inhibition, therefore, can suppress several forms of cell death, and it can also modulate the generation of various pro-inflammatory mediators [1].

Sepsis is defined as life-threatening organ dysfunction which develops in response to a dysregulated host response to infection [7]. Sepsis and septic shock remain important causes of morbidity and mortality worldwide. Evaluation of sepsis-related deaths among the underlying causes of death in the Global Burden of Diseases, Injuries, and Risk Factors Study (GBD), 2017, estimated 48.9 million cases of sepsis worldwide and 11 million sepsis-related deaths, representing 19.7% of all global deaths [8]. In Brazil, one-third of beds in intensive care units are occupied by patients with sepsis, and more than half of these patients die [9]. Similarly, in Switzerland, sepsis has a high mortality rate, and medical costs and loss of productivity represent a considerable burden to Swiss society [10]. Recent studies have shown that successful recovery depends on harmony between pro-and anti-inflammatory responses with inhibited, preserved, and increased cellular functions, a biologically relevant modulation that aims to control inflammation while preserving the anti-infective response [7,11].

A significant body of work has demonstrated the role of PARP1 overactivation in various inflammatory and infectious diseases, with nitric oxide (NO), reactive oxygen species (ROS), and peroxynitrite (ONOO^−^) being important triggers in this process [1,2,12,13]. Patients with sepsis produce ROS, NO, and ONOO^−^ at an increased rate [14,15,16,17,18,19]. These molecules play an important role as triggers for DNA single-strand breakage, a mechanism by which PARP1 is activated [1,12,16].

Sepsis and other forms of critical illness are closely associated with PARP1 activation [1,4,20,21,22]. Notably, we have previously demonstrated PARP1 activation in myocardial samples from patients with sepsis, with a significant correlation with the degree of cardiac dysfunction [23], and in the skeletal muscle of pediatric patients with burn injury [24].

The benefits of olaparib and other clinically approved PARP inhibitors have already been demonstrated in several experimental models of human diseases, including neurodegeneration and neuroinflammation, acute hepatitis, skeletal muscle disorders, aging, and acute ischemic stroke. Protection from the deterioration of the blood–brain barrier, restoration of the cellular levels of NAD^+^, improvement of mitochondrial function and biogenesis, and reduction of oxidative stress and pro-inflammatory mediators, such as tumor necrosis-alpha (TNF-α), IL1-β, IL-6, and vascular cell adhesion protein 1 (VCAM1), have been described [1,4,13].

To support the emerging concept of repositioning clinically used PARP inhibitors for the experimental therapy of septic shock, further data are required on the preclinical efficacy and safety of PARP1 inhibitors in experimental conditions that mimic sepsis or septic shock. Thus, here we have evaluated the effect of PARP1 inhibition in peripheral blood cells (PBMCs) obtained from healthy human volunteers following exposure of the cells to bacteria, bacterial pro-inflammatory components, or oxidative stress and assessed the effect of the PARP inhibitor on cell functions related to host response to infection: phagocytosis, ROS and NO generation, microbicidal activity, and cytokine production.

The data presented in the current article demonstrate that olaparib, in clinically relevant concentrations, exerts cytoprotective effects and modulatory effects on pro-inflammatory mediator production without adversely affecting cell viability or the ability of the immune cells to counteract pathogens. These data support further translational work towards the repurposing of PARP inhibitors for the experimental therapy of sepsis.

## 2. Materials and Methods

### 2.1. Healthy Volunteers and PBMC Preparations

Blood samples were collected from healthy volunteers between December 2017 and February 2022. Healthy human volunteers 18–50 years of age were used, with 59% of the volunteers being women and 41% being men. PBMCs were prepared as described in [18,19] by the Ficoll density gradient method (Ficoll-Paque plus) and suspended in RPMI 1640 medium (Sigma, St. Louis, MO, USA) supplemented with 10% fetal calf serum, 10 IU/mL penicillin, 10 μg/mL streptomycin (Gibco, Gaithersburg, MD, USA), and 200 mM L-glutamine (Sigma). Cell viability and count were determined with trypan blue using a hemocytometer.

### 2.2. Olaparib, PJ-34, LPS, Gram-Negative, and Gram-Positive Bacteria 

Olaparib and PJ-34 were purchased from Sigma-Aldrich (St. Louis, MO, USA). LPS from *Salmonella abortus equi* was a generous gift from Dr. C. Galanos (Max Planck Institute of Immunobiology, Freiburg, Germany). *Pseudomonas aeruginosa* (ATCC27853) and *S. aureus* (ATCC 25923) were purchased from Oxoid Limited (Basingstoke, Hampshire, UK).

### 2.3. Cell Viability Assays

To assess the cellular toxicity of olaparib and PJ-34, an apoptosis/necrosis detection assay was performed using peripheral blood mononuclear cells (PBMCs) from healthy individuals. Cells (2 × 10^6^/mL)were incubated in nonadherent tubes containing RPMI medium supplemented with 10% human AB serum in the presence of different concentrations of olaparib (0.1, 1, 10, 100, and 1000 µM) for 24 h in a 5% CO_2_ incubator at 37 °C. The cells were harvested, washed with phosphate-buffered saline (PBS), suspended in 100 µL of binding buffer, and labeled with 5 µL Annexin V and 5 µL 7-AAD (BD Biosciences, San Jose, CA, USA). After incubation for 15 min in the dark, 400 µL of binding buffer was added to each tube, and flow cytometry was performed.

### 2.4. PARP1 Detection and Detection of PARylation

The expression of PARP1 and the presence of protein-bound PAR polymers were evaluated using Western blotting. PBMCs were stimulated with *S. aureus* (4.8 × 10^8^ cells/mL) for 24 h to induce PARP1 activation. The effects of olaparib (1 and 10 μM) in preventing protein PARylation were evaluated. Proteins were extracted, and 30 µg of cell lysate was resolved by 10% SDS-PAGE. Protein expression was evaluated by Western blotting using an anti-PARP1 rabbit polyclonal antibody at 1:1000 dilution (cat. no. 9542S; Cell Signaling Technology, Beverly, MA, USA) and an anti-PAR rabbit polyclonal antibody at 1:1000 dilution (4336-BPC; Trevigen, Gaithersburg, MD, USA) overnight at 4 °C. β-actin mouse monoclonal antibody (cat. no. 47778; Santa Cruz Biotechnology, Dallas, TX, USA) was used as the loading control at 1:5000 dilution. The secondary anti-rabbit IgG, HRP-linked antibody purchased from Cell Signaling Technology (cat. no. 7074S) was diluted at 1:20,000. The secondary goat anti-mouse IgG-HRP (sc-2005; Santa Cruz Biotechnology, Dallas, TX, USA) was diluted at 1:250,000. Secondary antibodies were incubated for 1 h at room temperature. Bound antibody signals were amplified with ECL Select (GE Healthcare, Chicago, IL, USA). Luminescent bands were visualized using an Alliance 2.7 photo documenter and analyzed using the UVIBAND MAX v15. 03b program (UVITEC, Cambridge, UK). The bands were quantified as arbitrary volume units.

### 2.5. Detection of Cellular NAD^+^ Levels in PBMCs

Cellular NAD^+^ levels were measured using an NAD^+^/NADH Colorimetric Assay Kit (ab65348, Abcam, Cambridge, UK). PBMCs (5 × 10^6^ cells) were incubated in nonadherent tubes in a 5% CO_2_ incubator at 37 °C under unstimulated conditions and with different concentrations of olaparib (1, 10, and 100 µM) for 4 h. H_2_O_2_ (Sigma-Aldrich, 250 µM) was added for the final 2 h. (The concentration of the oxidant was selected based on pilot studies; we aimed to achieve an approximately 50% decrease in cellular NAD^+^ and ATP levels). After washing, cell lysis was performed using protease inhibitor extraction buffer according to the manufacturer’s instructions. The samples were centrifuged at 16,000× *g* for 5 min at 4 °C. The supernatant was collected and stored on ice. Six microliters of the sample was removed for subsequent protein quantification. The remainder was transferred to a 10 kDa Spin Column supported on a microtube and centrifuged at 10,000× *g* for 30 min at 4 °C to remove proteins and enzymes that could degrade NAD^+^. The filtrate was subjected to rapid freezing in liquid nitrogen and stored at −80 °C. Protein quantification was performed by the colorimetric method using a bovine serum albumin standard curve at 550 nm using a Multiskan Ex device (Thermo Fisher, Waltham, MA, USA). NAD^+^ was measured using a colorimetric method as described in [21].

### 2.6. Detection of Cellular ATP Levels in PBMCs

ATP levels were measured using the ATP Fluorometric Assay Kit (ab83355, Abcam) from the cell extracts generated in Section 2.5 (see above) using a fluorometric method in a 96-well plate with a black wall and a transparent bottom, according to the manufacturer’s instructions. Fluorescence readings (excitation: 535 nm; emission: 587 nm) were performed using a Synergy H1 microplate reader (Biotek Instruments, Winooski, VT, USA). The results are reported as pmol NAD^+^ or ATP/μg protein.

### 2.7. Mitochondrial Membrane Potential (MMP) Measurement

MMP was measured by flow cytometry using a JC-10 Assay Kit (ab112133; Abcam). PBMCs (2.5 × 10^6^/mL)were incubated in nonadherent tubes in a 5% CO_2_ incubator at 37 °C with different concentrations of olaparib (0.1, 1, and 10 µM) for 4 h. H_2_O_2_ (250 µM) was added for the last 2 h. As a positive control for the reaction, a tube was prepared wherein 5 µM of carbonyl cyanide 4-(trifluoromethoxy) phenylhydrazone (FCCP; Abcam) was added during the last 10 min. The cells were washed with PBS to remove the culture medium and centrifuged at 800× *g* for 5 min at 23 °C. The supernatant was discarded, and the cells were suspended in 1 mL of sterile PBS and transferred to labeled cytometry tubes. After centrifugation, 500 µL of JC-10 reagent was added to each tube, and the tubes were returned to the CO_2_ incubator for another 10 min. The samples were kept at room temperature and protected from light until flow cytometry. The acquisition time limit was 1 h, as per the kit’s recommendation. The intensity ratio of FL1/FL2 was used to monitor the MMP change induced by H_2_O_2_.

### 2.8. PBMC Culture and Measurement of Secreted Cytokines

PBMCs (2.5 × 10^6^/mL) were incubated in nonadherent tubes in a 5% CO_2_ incubator at 37 °C in the presence (100 ng/mL) of LPS; olaparib (0, 10, or 100 µM) was added 30 min before or after exposure to (100 µM) LPS. After incubation, the tubes were gently vortexed to homogenize the cell suspension and centrifuged at 400× *g* for 10 min at 4 °C. The supernatant was stored at −80 °C and tested for the presence of IL-10, IL-8, TNF-α, and macrophage inflammatory protein-1 alpha (MIP-1α) using a cytometric bead array (CBA; BD Bioscience), and it was tested for the presence of IL-6 using ELISA.

### 2.9. Measurement of ROS and NO Production

Whole blood was incubated in the absence or presence of olaparib (0.1, 1, and 10 μM) for 4 h. ROS and NO generation were measured constitutively and after stimulation with heat-killed *S. aureus* for 30 min. ROS and NO levels in monocytes and neutrophils were quantified by measuring the oxidation of 2,7-dichlorofluorescein diacetate (DCFH-DA; Sigma-Aldrich) and 4-amino-5-methylamino-2,7-difluorofluorescein diacetate (DAF-FMDA; Invitrogen, Carlsbad, CA, USA), respectively, using flow cytometry. Briefly, the tubes from each sample were incubated in the presence of 0.06 mM DCFH-DA or 0.01 mM DAF-FMDA in a 37 °C shaking water bath for 30 min. After incubation, 2 mL of 3 mM EDTA (Sigma-Aldrich) or PBS was added to each tube for ROS and NO determination, respectively, and the mixture was centrifuged at 800× *g* for 5 min at 4 °C. Erythrocytes were lysed in hypotonic saline, and the pellets were incubated with 5 μL of CD14 antibody (BV-711; BD Bioscience) at room temperature for 15 min in the dark. Two milliliters PBS was added to each tube, and the mixture was centrifuged at 800× *g* for 5 min at 4 °C. The supernatants were discarded, and the pellets were resuspended in 300 μL PBS for flow cytometry analysis.

### 2.10. Measurement of Phagocytosis

Phagocytosis was measured in monocytes and neutrophils using pHrodo™ Green *E. coli* BioParticles™ conjugated to fluorescein isothiocyanate (FITC) Cat. P35381 (Invitrogen, Carlsbad, CA, USA). Whole blood (with heparin anticoagulant), distributed in two sets of polypropylene tubes (control tubes and test tubes), was incubated in the absence or presence of different concentrations of olaparib (1–100 µM) in a 5% CO_2_ incubator at 37 °C for 4 h and stored on ice for 10 min. The tubes were kept on ice, and 20 μL pHrodo BioParticles^®^ was added to each tube, followed by brief vortexing to homogenize each suspension. The test tube set was placed in a 37 °C water bath in the dark for 30 min while the control tube set remained on ice. After incubation, the test tubes were placed on ice. Lysis of red blood cells was performed. The cells were suspended in washing buffer and centrifuged at 350× *g* for 5 min at room temperature. The supernatant was discarded. Surface labeling with anti-CD14 antibody (BV-711) was performed, followed by incubation for 15 min in the dark at room temperature. Cells were washed with Macs buffer, suspended in 500 µL of fixation buffer, and incubated for 30 min at 4 °C in the dark. The tubes were centrifuged, and the supernatant was discarded. The cells were suspended in wash buffer for further analysis by flow cytometry. The data were plotted as a histogram. The geometric mean of the fluorescence intensity (MGFI) was related to the detection of pHrodo™ Green *E. coli* BioParticles™ conjugate. The MGFI value of each tube in the control tube set (ice) was subtracted from that of its respective tube in the test tube set (37 °C).

### 2.11. Measurement of Bacterial Killing

To determine the influence of olaparib on the microbicidal ability of cells, *S. aureus* (ATCC 25213) was cultured in Tryptic Soy Broth (Difco, Detroit, MI, USA) for 18 to 24 h at 37 °C. The concentration of bacteria was determined by absorbance. PBMCs and PMNs (1 × 10^6^) were pre-incubated in the absence or presence of different concentrations of olaparib (0.1–10 µM) in a 5% CO_2_ atmosphere at 37 °C for 1 h. The cells were washed with PBS and incubated with 2 × 10^6^ *S. aureus* for 3 h at 37 °C. PBMCs and PMNs were lysed with 500 µL of 0.2% Triton X-100, sonicated for 2 min, and kept at room temperature for 15 min. The samples were diluted, seeded in Petri dishes containing tryptic soy agar (Difco), and incubated at 37 °C. Colony number was enumerated as colony-forming units (CFU/mL) after 24 h. Control refers to colony growth in the absence of PBMCs or PMNs.

### 2.12. Flow Cytometry

Cell viability, detection of ROS and NO, MMP, phagocytosis, and measurement of cytokines in the supernatant by CBA were performed by multiparameter flow cytometry using an LSRFORTESSA flow cytometer (BD Biosciences). Event acquisition was performed using the FACSDiva software. Analyses were performed using the FlowJo or FCAP Array 3.0 software packages (BD Biosciences).

### 2.13. Statistical Analysis

Data are presented as representative blots or mean ± SEM of experiments performed on at least *n* = 3 experimental days. ANOVA followed by Tukey’s multiple comparisons post hoc test was used. A *p* < 0.05 was considered statistically significant.

## 3. Results

### 3.1. Clinically Relevant Concentrations of Olaparib Do Not Alter Human Lymphocyte Viability 

**Figure 1:**
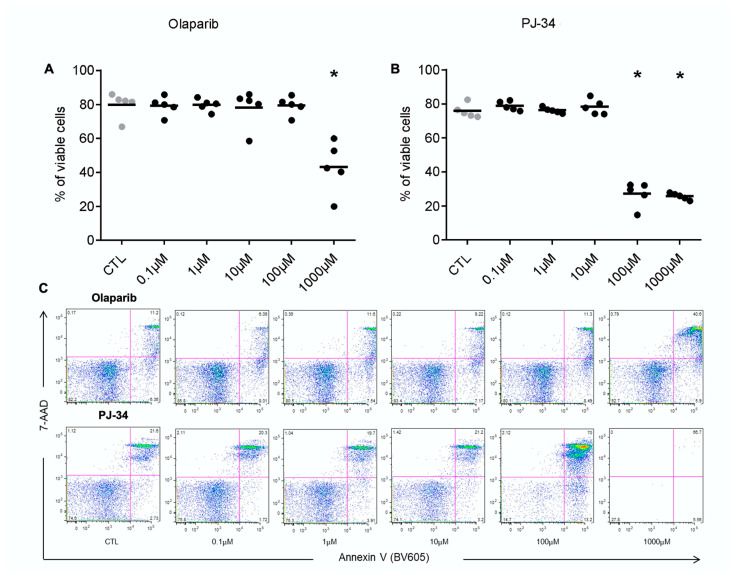
Effect of olaparib and PJ-34 on PBMC viability. (**A**,**B**) Analysis of cell viability in PBMCs from five healthy individuals at different concentrations of olaparib and PJ-34 (0.1–1000 µM). (**C**) Representative dot plots. Phosphatidylserine externalization was detected via Annexin V staining, and a change in cell membrane integrity (resulting in increased 7-ADD dye uptake) was detected via 7-ADD staining. “Viable cells” in (**A**,**B**) are defined as the portion of cells that were negative for both Annexin and 7-ADD. * *p* < 0.01 shows significant decrease in cell viability compared to the control (ANOVA followed by Tukey’s test).

Cell viability was not affected by olaparib at 0.1, 1.0, 10, and 100 µM, while at the highest concentration of 1000 µM—which is substantially higher than the clinically relevant concentration of the inhibitor—an inhibitory effect was noted. Cell viability was unaffected by the earlier-generation PARP inhibitor PJ-34 at 0.1, 1.0, and 10 µM, but it was suppressed at 100 and 1000 µM (Figure 1).

Considering the lower toxicity of olaparib, coupled with the fact that this PARP inhibitor is currently in clinical use, olaparib was exclusively used in the subsequent experiments.

### 3.2. Olaparib Inhibits Protein PARylation in Human PBMCs in a Concentration-Dependent Manner

**Figure 2:**
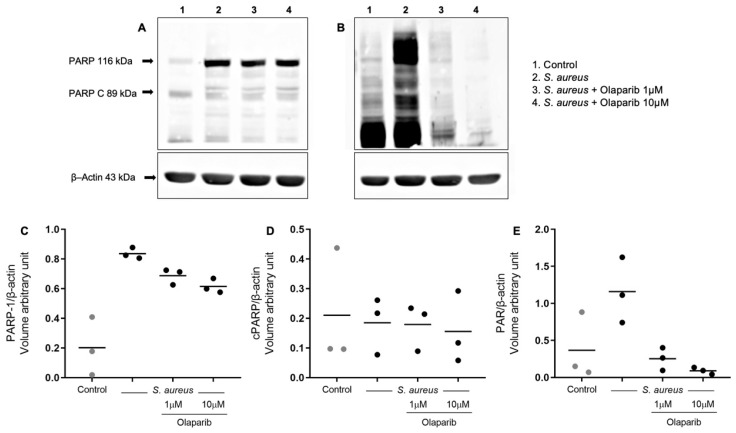
Effect of olaparib on PARP1 expression and protein PARylation in human PBMCs stimulated with *S. aureus*. Effects of olaparib (1 and 10 µM) on protein expression of PARP1 and cleaved PARP1 (**A**) and on the increase in PARylation of PARP1 (**B**) in PBMCs from healthy individuals in unstimulated conditions (control, CTL) and after *S. aureus* stimulation. Bands represent one individual from three volunteers. Densitometric analyses of the effects of olaparib on the protein expression of PARP1 (**C**), cleaved PARP1 (**D**), and PARylation of PARP1 (**E**). Each plot represents three healthy individuals using β-actin as a loading control.

PARP1 expression in PBMCs from healthy individuals was increased after stimulation with *S. aureus*. This enzyme induction was not affected by olaparib (1 and 10 µM) (Figure 2A,C). There was also evidence for PARP1 cleavage, which is the result of cellular caspase activity; the amount of cleaved PARP was similar in all of our experimental conditions (Figure 2A,D). PARP1 activation was evidenced by PAR polymer detection at the 116 kDa protein band, which is due to PARP1 protein PARylation (i.e., auto-PARylation of the enzyme that produces these polymers). An increase in PARylation was observed after stimulation with *S. aureus*, likely representing increased PARP1 catalytic activity, coupled with increased PARP1 protein expression. Olaparib (1 and 10 µM) reduced *S. aureus*-induced PARylation in a concentration-dependent manner (Figure 2B,E).

### 3.3. NAD^+^ and ATP Depletion Is Prevented, and H_2_O_2_-Induced Mitochondrial Membrane Depolarization Is Attenuated by Olaparib in PBMCs Subjected to Oxidative Stress

**Figure 3:**
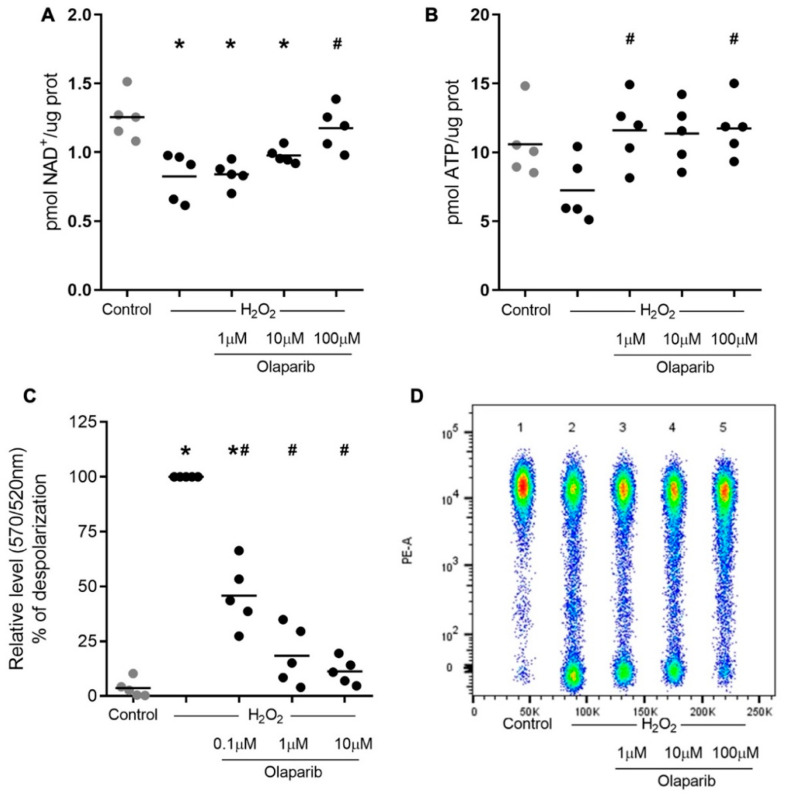
Effect of olaparib on NAD^+^ and ATP levels of human PBMCs exposed to oxidative stress. PBMCs from five healthy volunteers were incubated for 4 h with increasing concentrations of olaparib (1–100 µM) and in the presence of H_2_O_2_ (250 µM) for 2 h. Protein was extracted, and NAD^+^ (**A**) and ATP (**B**) levels were quantified by colorimetric and fluorometric assays, respectively. Values are expressed in pmol NAD^+^ or ATP/ug protein. Mitochondrial membrane potential (MMP) measurements (**C**) were conducted in lymphocytes; MMP is expressed as the FL-1/FL-2 GMFI ratio. (**D**) Dot plots representative of concatenating analyses from five individuals. * *p* < 0.01 compared to the control condition. ^#^ *p* < 0.05 compared to the H_2_O_2_ condition. (ANOVA followed by Tukey’s test).

Consistent with its PARP-activating effect, H_2_O_2_ caused a significant decrease in intracellular NAD^+^ levels. This effect was prevented by olaparib in a concentration-dependent manner (1–100 µM) (Figure 3A). A decrease in intracellular ATP levels was also induced by H_2_O_2_. Olaparib prevented this response as well (Figure 3B).

H_2_O_2_ also induced depolarization of the mitochondrial membrane of the cells, and olaparib (0.1–10 µM) also prevented this response; at 10 µM, olaparib restored this parameter to healthy control levels (Figure 3C). A representative dot plot of concatenating analyses from five individuals is shown in Figure 3D.

### 3.4. Olaparib Modulates Cytokine Secretion in Human PBMCs Stimulated with LPS

**Figure 4:**
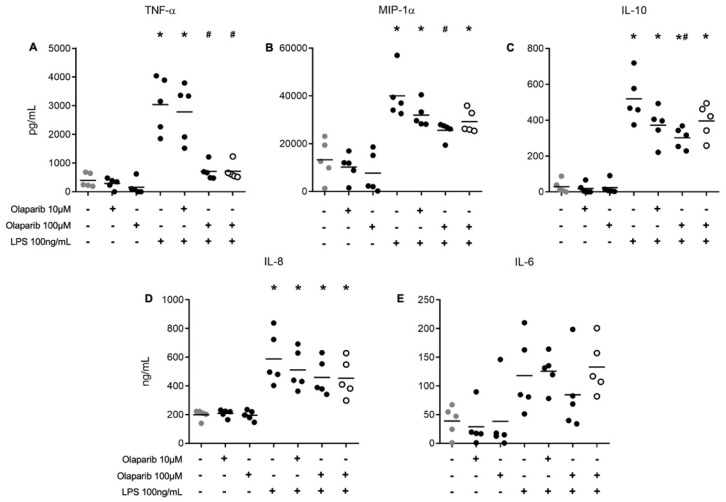
Measurement of cytokines secreted by human PBMCs. PBMCs from five healthy volunteers were incubated for 24 h with or without LPS (100 ng/mL). Cultures were untreated, or olaparib (10 or 100 µM) was added 30 min before exposure to 100 µM LPS (closed symbols). The last group (open symbols) represents the results of the experiments when olaparib (100 µM) was administered 30 min after LPS (as opposed to the pretreatment paradigm applied in the rest of the protocol). Culture supernatants were examined at 24 h. The results for IL-6 were obtained by ELISA assay (ng/mL). The results for TNF-α (**A**) MIP-1α (**B**), IL-10 (**C**), and IL-8 (**D**) were obtained by the CBA assay (pg/mL) and by ELISA for IL-6 (**E**). * *p* < 0.05 compared to the control condition. ^#^ *p* < 0.05 compared to the LPS 100 ng/mL condition (ANOVA followed by Tukey test).

Measurement of the cells’ supernatants for secreted cytokines revealed that, in general, cells produced multiple cytokines—including TNF-α, MIP-1α, IL-10, IL-8, and IL-6—after stimulation with 100 ng/mL LPS (Figure 4). Levels of TNF-α, MIP-1α, and IL-10 were reduced in the presence of 100 μM olaparib, but to different degrees: while TNF-α levels were reduced nearly to baseline control levels, the effect of olaparib on MIP-1α and IL-10 levels was only partial. In addition, no significant effect of olaparib was seen on IL-8 or IL-6 levels. These data indicate that olaparib exerts a selective modulatory effect on cytokine production; overall, it tends to inhibit pro-inflammatory cytokines more than anti-inflammatory ones. Olaparib did not affect the low levels of basal cytokine production in unstimulated cells. Olaparib pre- vs. post-treatment (30 min before or after the LPS stimulus, tested only with 100 µM of the PARP inhibitor) produced similar effects (Figure 4).

### 3.5. Olaparib Does Not Interfere with Pathogen Eradication by Human Leukocytes

Monocytes and neutrophils showed increased ROS and NO production after stimulation with *S. aureus* (approx. 2-fold and 11-fold increases, respectively, *p* < 0.05). *S. aureus*-stimulated ROS and NO production was unaffected by olaparib (0.1–10 µM) (Table 1).

**Figure 5:**
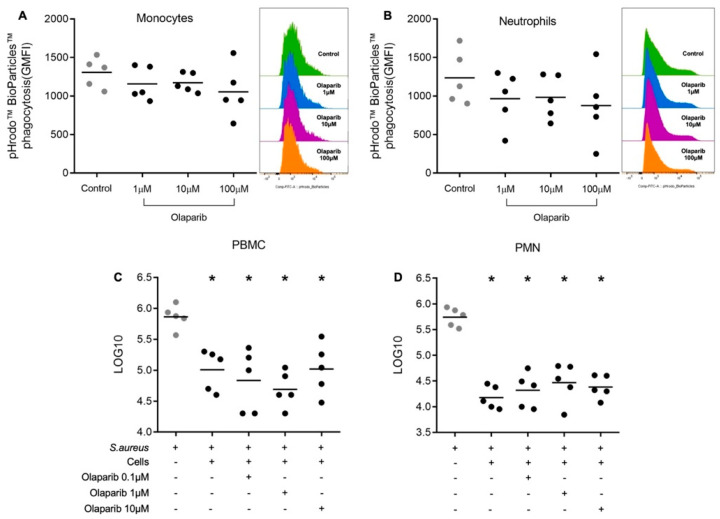
Effect of olaparib on *S. aureus* killing by human leukocytes. Phagocytosis was measured in monocytes (**A**) and neutrophils (**B**). Whole blood from five healthy individuals was pre-incubated with olaparib at different concentrations (1–100 μM) for 4 h, followed by incubation with pHrodo™ Green *E. coli* BioParticles™. Data are shown as geometric mean fluorescence intensity. Killing activity against *S. aureus* was measured in PBMCs (**C**) and PMNs (**D**) from five healthy volunteers in the presence of increasing concentrations of olaparib (0.1–10 µM). Cells were pre-incubated with the PARP inhibitor for 1 h, and then *S. aureus* was added to the cells. The control condition refers to the growth of *S. aureus* colonies in the absence of PBMCs or PMNs. Results are expressed as log 10 CFU. * *p* < 0.05 compared to the control condition (ANOVA followed by Tukey’s test).

Olaparib (1–100 μM) did not impair the phagocytic activity of monocytes and neutrophils (Figure 5A,B). PBMCs and PMNs from healthy individuals were effective in eradicating *S. aureus*. This effect was maintained in the presence of different concentrations of olaparib (0.1–10 µM) (Figure 5C,D); this finding is consistent with the lack of effect of the PARP inhibitor on the production of ROS and NO, which are known to play a significant role in bacterial elimination responses.

## 4. Discussion

Olaparib was the first inhibitor approved for clinical use. Its approval in 2014 created a new perspective on the use these inhibitors in the treatment of non-oncological diseases—as reviewed in [4,13]. The use of olaparib in cancer therapy is mainly based on its specific action in cells with BRCA1 and BRCA2 gene mutations. Cells with the mutations accumulate double-strand breaks in the presence of PARP1 inhibitors, resulting in cell death. In this scenario, its use is limited to a specific group of patients and can be associated with other cancer drugs. An important point that must be considered is that olaparib is administered at relatively high doses in cancer therapy. However, several benefits in modulating cellular responses have been observed in different non-oncological disease models using lower doses of olaparib, including sepsis [1,4,21].

In this context, our findings show that olaparib in the concentration range of 1–100 µM is not detrimental to the viability of human peripheral blood cells but protects against H_2_O_2_ induced toxicity, modulating inflammatory mediator secretion (predominantly by inhibiting pro-inflammatory cytokines); at the same time, the PARP inhibitor does not appear to interfere with the cells’ ability to execute phagocytosis or to eliminate bacteria. The concentrations of olaparib used in the various assays were based on preliminary experiments. In experiments where substantial or near-complete effects of olaparib were already noted in the concentration range of 1–10 µM (e.g., ATP and NAD^+^ levels, MMP), higher concentrations of olaparib were not examined. However, in experiments where the effect of olaparib in the 1–10 µM concentration range was only partial (e.g., on cytokine responses) or where no effect was observed (e.g., on phagocytosis), we also included a higher (100 µM) concentration in the experiments. We are well aware that this latter concentration of olaparib is substantially higher than the plasma levels of olaparib in oncological patients. For instance, oral administration of 300 mg olaparib yields approximately 20 µM peak plasma olaparib concentration [25]. As discussed previously [4,13,21], we anticipate that repurposing studies of olaparib in sepsis will require lower doses than the doses used in oncology, and consequently, the plasma levels achieved will also be lower that the plasma levels in oncological patients. At these lower plasma levels (1–10 µM), based on the current results, we can anticipate cytoprotective and beneficial cellular bioenergetic effects, as well as partial modulatory effects on the generation of various inflammatory mediators, but no adverse effects on phagocytosis or bacterial killing.

Interestingly, we observed an increase in PARP1 protein expression when the cells were incubated with *S. aureus*. In our control cell preparations, the expression of PARP1 was detectable but relatively low, and there was also a secondary band, consistent with a partial PARP cleavage. (This “baseline” PARP1 cleavage in freshly isolated PBMCs has also been observed by other investigators, e.g., ref. [26]; its underlying mechanism remains to be further explored). After *S. aureus* incubation, the PARP1 signal was increased, while the lower PARP bands did not change substantially. PARP is known to be regulated at the level of transcription, as well as protein stability and degradation [1,2]. The current project was not designed to determine the mechanisms responsible for the increased PARP1 expression. Instead, the goal was to evaluate the effects of olaparib. Indeed, at low concentrations of 1 and 10 µM, as expected, olaparib did not interfere with the increase in PARP1 protein expression induced by *S. aureus*. As expected, olaparib inhibited *S. aureus*-stimulated protein PARylation in a concentration-dependent manner. 

PARP1 activation has been observed in both experimental and clinical models of acute and chronic disease [1,2]. Resistance to inflammation has been observed in PARP1 knockout mice subjected to various forms of injury including reperfusion and inflammation [1,2]. Also, PARP1 activation plays a significant role in the pathogenesis of ARDS [27,28,29]. Pediatric patients with serious burn injuries reportedly presented with PARP1 activation, as demonstrated by increased PARylation in endothelial cells and leukocytes from muscle tissue [24]. PARP1 activity has been significantly associated with myocardial dysfunction in patients with septic shock [23]. PARP1 activation was previously observed in the nuclei of cardiomyocytes after transplantation [30] and in mononuclear cells from patients with myocardial infarction [31], as well as in septic shock patients with myocarditis [32]. In another study, olaparib protected against acute hepatitis by reducing overall protein PARylation, improving liver function, and reducing the expression of inflammatory genes in a liver injury model induced by endotoxin injection [33].

As previously reported regarding other models and other PARP inhibitors including PJ-34 [34,35,36,37], we observed a significant, PARP1-related decrease in NAD^+^ and ATP levels induced by H_2_O_2_ in PBMCs. These effects were prevented by olaparib in a concentration-dependent manner. The previously demonstrated PARP1-dependent H_2_O_2_-induced depolarization of the lymphocyte mitochondrial membrane [38] was also counteracted by olaparib, and at the highest concentration used, it was restored to healthy control levels. A significant increase in PARP1 expression, PARylated proteins, and ATP depletion resulting in necrosis has been observed in various cell types challenged with H_2_O_2_ [39,40]. In addition to reducing ATP levels, NAD^+^ consumption also has implications for reducing the rate of glycolysis, which several studies have supported as a source of energy and modulation of immune functions [11,41,42,43,44]. In the experiments involving *S. aureus* exposure, the current study focused on the effect of olaparib on pro-inflammatory mediator production; in these experiments, we did not measure the cellular levels of ATP and NAD^+^. Whether such changes occur and whether there is any functional interlink between the bioenergetic effects of PARP1 activation and the role of PARP1 in modulating cellular inflammatory mediator production remains to be investigated in future studies.

Supporting our results from PBMCs, olaparib induced a significant decrease in PARP1 activity and recovery of NAD^+^ levels, and it increased cell viability in an acute pancreatitis model and human monocytic lineage cells subjected to oxidative stress. In vivo, olaparib improves organ function and prolongs survival in experimental septic shock without adverse effects on DNA integrity [21,45]. Mitochondrial dysfunction plays an important role in sepsis pathophysiology. The direct effect of PARP1 overexpression on NAD^+^ and ATP levels results in mitochondrial dysfunction associated with cellular bioenergetic deficits. Bioenergetic dysfunction of this important cellular organelle is related to multiple organ dysfunction in sepsis and may be crucial for the severity and outcome of septic shock [4,46].

In a previous study by our group, we evaluated the interaction between the activation of a pathogen via TLRs and danger signal receptors (NOD-like receptors (NLRs) with oxidative metabolism and oxidative phosphorylation in patients with sepsis. We observed that genes related to mitochondrial oxidative phosphorylation of complexes I, IV, and V were downregulated, as were those involved in mitochondrial ROS elimination, including superoxide dismutase (SOD)1 and SOD3, catalase, peroxiredoxin (PRDX)-3 and 4, and thioredoxin reductase (TXNDRD) 1 and 2. Thus, mitochondrial dysfunction and components of oxidative phosphorylation are among the most altered canonical pathways in non-surviving patients, reinforcing the relationship between the maintenance of mitochondrial stability and the recovery of patients with sepsis [47]. Accordingly, PARP1 inhibition was found to be beneficial for mitochondrial function and left ventricular function in a study that evaluated the role of PARP1 in the maintenance of mtDNA-dependent mitochondrial function in Chagas disease [48]. Significant depletion of NAD^+^ and/or ATP and altered mitochondrial function have been consistently observed in different models of shock and sepsis. These alterations are partially reversible by PARP inhibitors, for example, in murine endotoxic shock studies [16]. Our results provide evidence for these protective effects in vitro using PBMCs.

PARP1 plays a major role in the inflammatory response. PARP1 and its catalytic activities induce macrophage activation, affecting the cell response to pathogen-associated molecular patterns, for example, LPS [1,6]. These effects on cytokine production are, at least in part, mediated through PARP1 and NF-ĸB interactions. Hassa and colleagues demonstrated that direct protein–protein interactions with both NF-ĸB subunits are necessary for their PARP1 coactivating functions [49]. Moreover, Bohio and colleagues demonstrated that tyrosine-phosphorylated PARP1 is required for PARylation of Re1A/p65 (the transcription activation subunit of NF-ĸB) and NF-ĸB-dependent expression of pro-inflammatory genes in murine RAW 264.7 macrophages, human monocytic THP1 cells, and mouse lungs [50]. In addition, PAR, a product of PARP, induces cytokine release in human and mouse macrophages through TLR2 and TLR4 activation, acting as an extracellular damage-associated molecular pattern that drives inflammatory signaling [5]. We observed that olaparib decreased the production of TNF-α, MIP-1α, and IL-10 in leukocytes from healthy individuals after in vitro LPS stimulation. Notably, these results were observed in the presence of a relatively high concentration (100 µM) of olaparib—a higher concentration than that needed to exert its protective effects on NAD^+^ consumption, ATP decrease, and mitochondrial depolarization.

The above findings are in line with multiple lines of in vivo data showing that olaparib can modulate inflammatory cytokine production in different animal injury models. In BALB/c mice treated with olaparib and subjected to burns, in addition to a reduced general inflammatory state in plasma, suppression of the production of inflammatory mediators that included IL-1β, TNF-α, and IL-6 was described [51]. In mice subjected to cecal ligation and puncture, olaparib reduced the plasma levels of several mediators, including cytokines, chemokines, and growth factors [21]. Olaparib also suppressed cytokine production in a model of reinfection and acute lung injury in BALB/c mice, coupled with a suppression of neutrophil infiltration and inhibition of oxidative stress [52]. Similar results were observed in a model of acute lung injury in mice elicited by intratracheal administration of endotoxin. A suppression of NF-ĸB-dependent gene expression, including IL-1β, TNF-α, and VCAM-1, was also observed with olaparib in a mouse model of acute respiratory distress syndrome [53]. Furthermore, olaparib treatment attenuated Th2 cytokine production in CD3/CD28-stimulated human CD4^+^ cells [54].

Various ROS and peroxynitrite (a reactive oxidant produced by the reaction of NO and superoxide) are potent triggers of DNA strand breakage, which activates PARP1, in turn leading to energy depletion and mitochondrial dysfunction culminating in cell necrosis [12,13,16]. We have previously evaluated monocyte functions by flow cytometry and observed preserved phagocytic activity, increased ROS and NO generation, and decreased production of inflammatory cytokines in sepsis and septic shock [18,19]. These events are likely desirable for reprogramming the activity of the immune system in a hostile environment [55,56]. Thus, we have tested whether olaparib would adversely affect the cellular responses to infection control (i.e., phagocytic activity and generation of ROS and NO) and intracellular bacterial killing. Olaparib did not suppress these responses, which is, indeed, the desirable outcome when considering the therapeutic repurposing of this agent for sepsis or septic shock.

## 5. Conclusions

In summary, our current study, investigating the effect of olaparib in peripheral blood leukocytes from healthy human volunteers, shows that olaparib at low concentrations (0.1–10 µM) inhibits protein PARylation, prevents oxidant-induced NAD^+^ and ATP depletion, and (at 100 µM) selectively modulates the production of various inflammatory mediators in response to LPS stimulation. These effects occur without any adverse effects of the PARP inhibitor on cells’ ability to eradicate pathogens via ROS and NO generation, or to perform monocyte and neutrophil phagocytosis, or to exert microbicidal activity. These results reinforce and support the emerging concept of repurposing clinically approved PARP inhibitors for the experimental therapy of non-oncological indications such as systemic inflammation, sepsis, and septic shock.

## Figures and Tables

**Table 1:** Effect of olaparib on ROS and NO production in monocytes and neutrophils. Cells were obtained from healthy volunteers (N = 5), and the assay was conducted without stimulus (control) or after *S. aureus* in the presence of olaparib (0.1–10 µM). Data are shown as the geometric mean fluorescence intensity (GMFI) of DCFH and DAF for ROS and NO, respectively.

	Monocytes Median (Percentiles 25–75)	Neutrophils Median (Percentiles 25–75)
**ROS**	1020 (836–1466)	693 (542–847)
Control	2053 (1804–2522)	8056 (7733–8586)
*S. aureus*	2430 (1680–3168)	9335 (6625–9806)
*S. aureus* + olaparib (0.1 µM)	2376 (1651–3050)	9635 (7528–10,122)
*S. aureus* + olaparib (1 µM)	1899 (1688–2634)	8147 (7021–1017)
*S. aureus* + olaparib (10 µM)	1020 (836–1466)	693 (542–847)
**NO**	467 (390–564)	448 (387–552)
Control	660 (616–921)	1066 (957–1288)
*S. aureus*	722 (587–1151)	1166 (976–1372)
*S. aureus* + olaparib (0.1 µM)	726 (700–1137)	1278 (1096–1446)
*S. aureus* + olaparib (1 µM)	467 (390–564)	448 (387–552)
*S. aureus* + olaparib (10 µM)	808 (675–1272)	1158 (1072–1696)

## Data Availability

The data presented in this study are available on request from the corresponding author.
